# Sex-related differences in medical cannabis use: A nation-wide database study

**DOI:** 10.1192/j.eurpsy.2023.256

**Published:** 2023-07-19

**Authors:** N. Yakirevich Amir, N. Treves, I. Reuveni, E. Davidson, O. Bonne, I. Matok

**Affiliations:** 1 Psychiatry, Hadassah University Medical Center; 2 school of pharmacy, Hebrew University of Jerusalem; 3 Anesthesiology, Hadassah University Medical Center, Jerusalem, Israel

## Abstract

**Introduction:**

Cannabis use is associated with mental illness among men and women, especially induction or exacerbation of psychosis, anxiety, and depression. Although safety and efficacy of cannabis in most medical conditions have not been established, use of medical cannabis is growing exponentially. In particular, albeit sex-related differences in the activity of the endocannabinoid system in animals and humans, differential effects of cannabis on men and women have rarely been sought.

**Objectives:**

To characterize patterns of use and adverse effects experienced by men and women using medical cannabis.

**Methods:**

Data from the Israeli national database of patients licensed to use medical cannabis in Israel from January 2014 to December 2021 was analyzed. The database includes indications for cannabis use, monthly cannabis quantities, Tetrahydrocannabinol (THC) and Cannabidiol (CBD) concentrations, and reports of adverse effects. Comparative statistics were used to evaluate the sex related differences.

**Results:**

161,644 persons (62% men) were issued a license to use medical cannabis during the study period. Men are significantly younger than women (50.5±19.1 vs. 56.5±18.4). The leading indications among both men and women are chronic pain (58% of men, 57% of women), symptoms related to oncological disease and chemotherapy treatment (21% of men, 24% of women) and post-traumatic stress disorder (9% of men, 6% of women). Men consume significantly higher monthly quantities at the beginning of treatment compared to women (31.6 gram vs. 29.3 gram) with a higher THC concentration (13.9% vs. 11.6%) and lower CBD concentration (5.3% vs. 6.7%). Over two years of use, there is an increase among both men and women in the amount and THC concentration, and a decrease in the CBD concentration. The differences between men and women remain significant throughout the whole period. Data on adverse effects are available for 28,629 men and 17,204 women (28.6% of men, 28.0% of women). Women report significantly more physical adverse effects (RR 1.48 [95%CI 1.39-1.57]), anxiety (RR 1.45 [95%CI 1.35-1.56]), depression (RR 1.36 [95%CI 0.95-1.96]) and derealization (RR 3.44 [95%CI 2.42-4.89]).

**Conclusions:**

Although the prevalence of medical conditions for which medical cannabis is indicated are similar for both genders, approximately 60% more men consume medical cannabis. While consuming lower cannabis amount and THC concentration, women report more physical and psychiatric adverse effects than men. Understanding the differences in usage patterns and adverse effects between men and women will enable more accurate policy determinations and more effective and safer treatment strategies.

**Disclosure of Interest:**

None Declared

